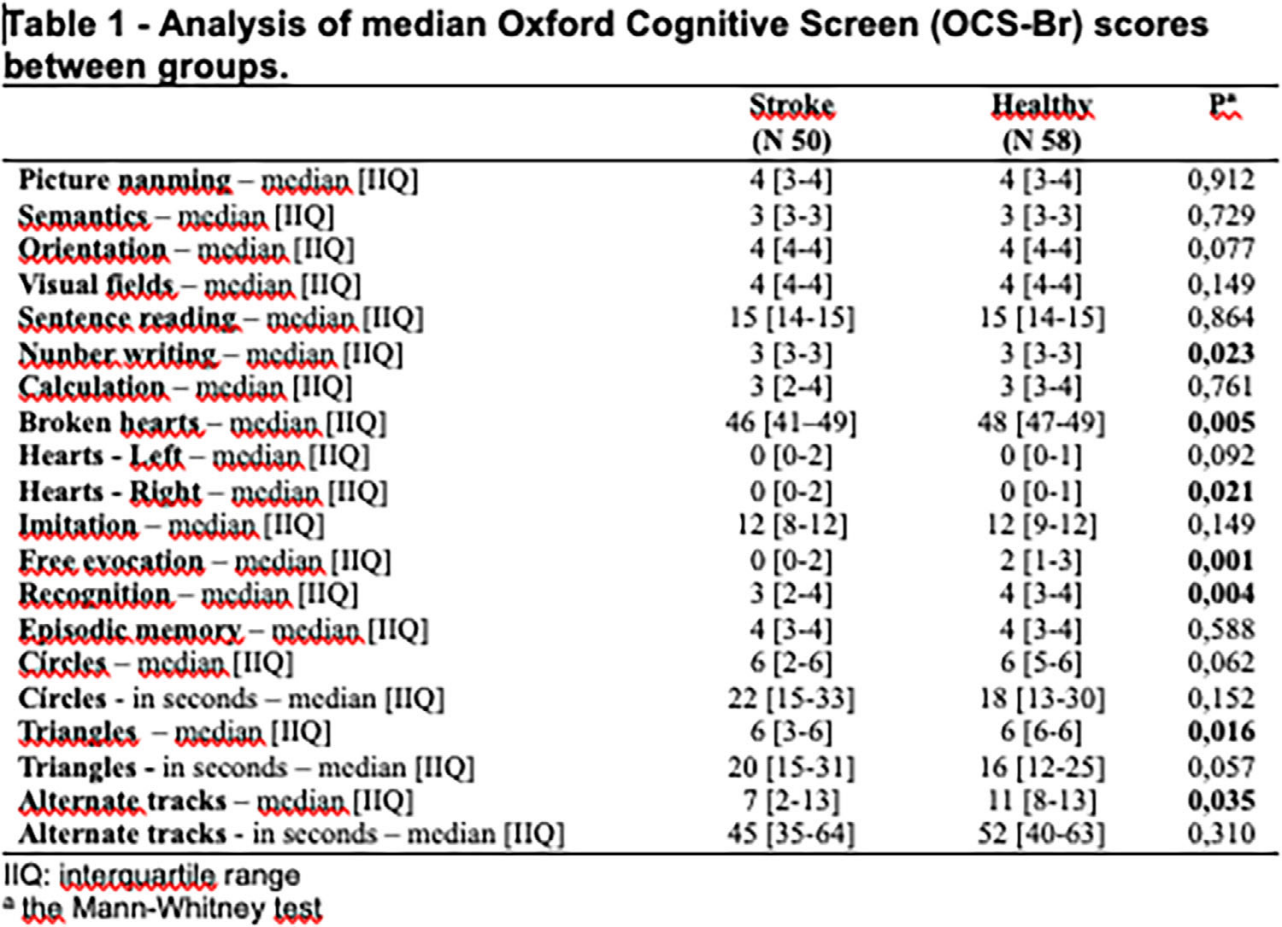# Oxford Cognitive Screen Brazilian Version: Assessment of Vascular Cognitive Impairment

**DOI:** 10.1002/alz.094850

**Published:** 2025-01-09

**Authors:** Claudia Cristina Ferreira Ramos, Caroline Suemi Ogusuku, Sonia Maria Dozzi Brucki, Ricardo Nitrini

**Affiliations:** ^1^ Universidade de São Paulo, São Paulo, São Paulo Brazil; ^2^ Hospital Santa Marcelina, São Paulo, São Paulo Brazil; ^3^ University of São Paulo School of Medicine, São Paulo, São Paulo Brazil; ^4^ Hospital das Clínicas da Faculdade de Medicina da Universidade de São Paulo, São Paulo, São Paulo Brazil

## Abstract

**Background:**

Cognitive impairment is very common in stroke patients and is rarely diagnosed. Cognitive deficits involving language functions, praxis, visuospatial, visuoconstructive skills and memory are prominent. The available cognitive assessment tests do not address some specific characteristics of stroke patients and have important limitations in relation to the most compromised cognitive domains.

**Method:**

Observational and descriptive study, with cognitively normal individuals and patients with a history of Cerebral Vascular Accident (CVA) from the Unified Health System of São Caetano do Sul and São Paulo in Brazil from September 2021 to July 2023.

**Result:**

108 participants were included in this evaluation, 50 (46.3%) from the stroke group and 58 (53.7%) from the healthy group. The average age was 67 years for the stroke group and 69 years for the healthy group. Our total average number of years of study was 9 years. When comparing the OCS‐Br scores between the groups, we found that there was a significant difference between the groups in writing tasks, executive functions (attention, change of strategy) and memory (Table 1). The comparison of scores on the OCS‐Br by classification of cognition was carried out with a group stratified between Cognitively Healthy and Cognitive Impairment With and Without Dementia, with a statistically significant result between the groups in orientation (p<0.013), visual field (p< 0.045), free evocation (p<0.002), recognition (p<0.010) and circles in seconds (p<0.008).

**Conclusion:**

With our results, the need for adequate monitoring and rehabilitation of post‐stroke patients becomes more evident. The advantages of the OCS‐Br are: focus on specific cognitive aspects of stroke, such as visual inattention and visual field testing; assessment in patients with aphasia and visual impairment and prognostic value to predict long‐term functioning